# Successful Management of Ventricular Tachycardia in an Adult Case of Unrepaired Pulmonary Atresia With Ventricular Septal Defect: A Case Report

**DOI:** 10.1016/j.cjcpc.2025.02.001

**Published:** 2025-02-27

**Authors:** Masao Matsuda, Suguru Nishiuchi, Makoto Miyake, Hirokazu Kondo, Toshihiro Tamura

**Affiliations:** a Department of Cardiology, Tenri Hospital, Tenri, Nara, Japan; b Department of Cardiology, Osaka City General Hospital, Miyakojima-ku, Osaka, Japan; c Division of Cardiology, Gunma Prefectural Cardiovascular Center, Maebashi, Gunma, Japan; d Congenital Heart Disease Center, Tenri Hospital, Tenri, Nara, Japan

**Patients with unrepaired pulmonary atresia with ventricular septal defect (PA/VSD), the extreme form of tetralogy of Fallot, typically face poor prognoses and unstable clinical conditions. The mechanisms underlying ventricular arrhythmias in PA/VSD and their optimal management remain unclear. However, we report the successful management of drug-resistant ventricular tachycardia in a 42-year-old woman with unrepaired PA/VSD through ablation, achieving a favorable clinical outcome. Functional substrate mapping and preoperative cardiac imaging proved valuable in identifying the arrhythmogenic substrate of the ventricular tachycardia. A flexible, multidisciplinary approach was essential during the perioperative period.**

Pulmonary atresia with ventricular septal defect (PA/VSD) is regarded as the extreme form of tetralogy of Fallot. It is frequently associated with hypoplastic pulmonary arteries, with pulmonary blood flow supplied by major aortopulmonary collateral arteries (MAPCAs). Until the early 1980s, patients with the absence of the main pulmonary artery were unable to undergo intracardiac repair and were limited to palliative procedures, such as the modified Blalock-Taussig shunt. However, since the late 1980s, the development of the unifocalization technique, which integrates pulmonary arteries with MAPCAs, has provided a means to enable antegrade blood flow from the right ventricle (RV) to the pulmonary arteries in such cases.^1^ Despite this advancement, the technique has a relatively short history, and there remain individuals aged 30-40 years today with unrepaired PA/VSD.

Although patients who survive to adulthood with unrepaired congenital heart disease (CHD) often maintain a relatively stable condition despite persistent cyanosis, the management of ventricular tachycardia (VT) is crucial. Once it occurs, VT can significantly reduce quality of life and may even be life threatening. However, there are only a limited number of reports addressing the mechanisms and management of VT in these patients.^2^

In a 42-year-old woman with unrepaired PA/VSD, we successfully identified the mechanisms of VT development using gadolinium-enhanced magnetic resonance imaging (MRI) and electrophysiological studies. The VT was effectively treated through catheter ablation. We herein report this successful case alongside a review of the relevant literature.

## Case

A 42-year-old woman was diagnosed with 22q11.2 deficiency syndrome and PA/VSD at birth. A conservative strategy was adopted because intracardiac repair was deemed unsuitable due to the absence of the main pulmonary artery and the unifocalization procedure was not available during her childhood. She presented with a grade 2/6 continuous cardiac murmur heard in the left interscapular region and cyanosis, with oxygen saturation (SpO_2_) ranging from 75% to 80%; however, she was clinically stable.

**Figure 1:**
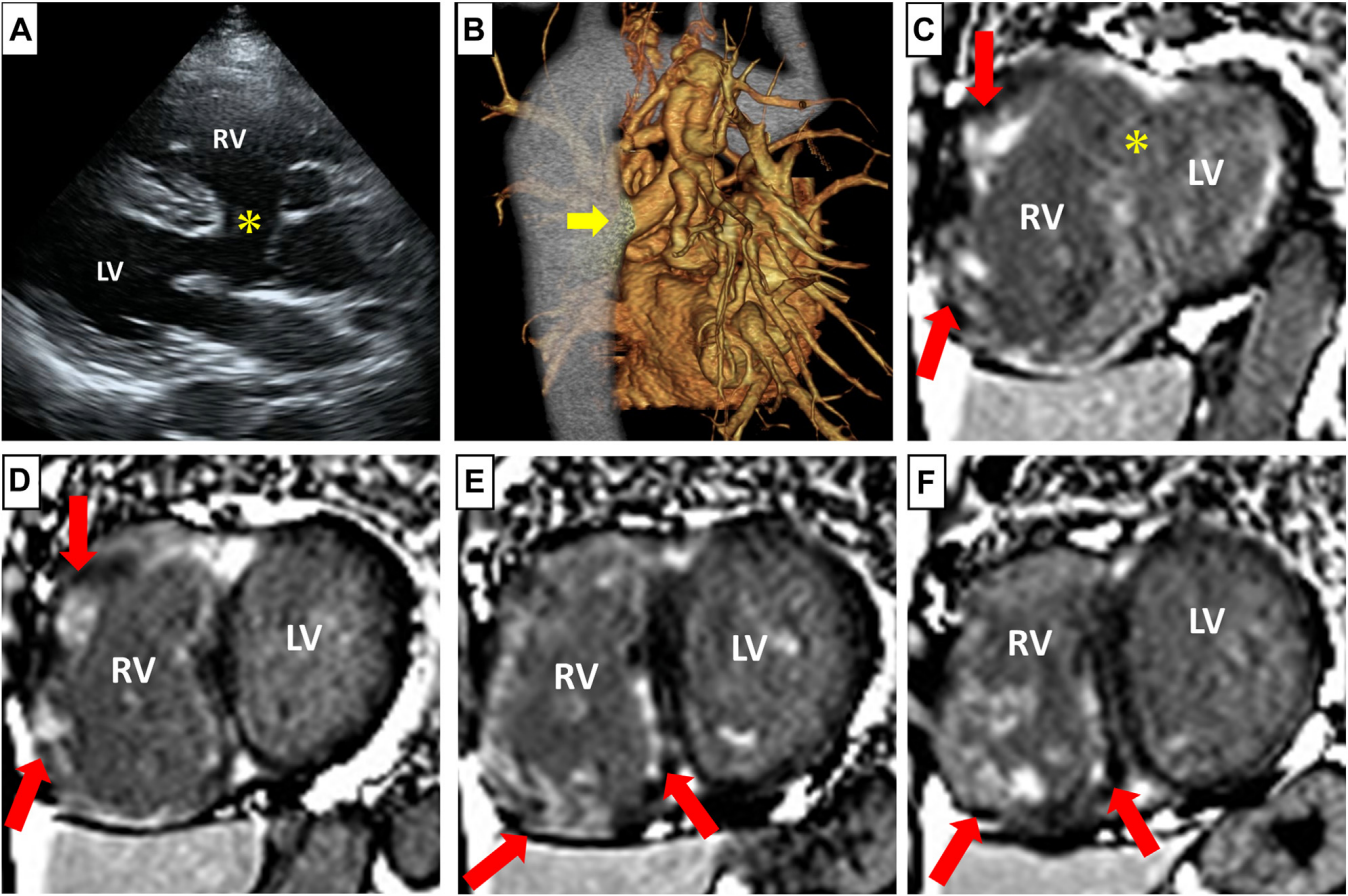
(**A**) Parasternal long-axis view on transthoracic echocardiography showing the VSD (**asterisk**), aortic override, and RV enlargement. (**B**) Right posterior oblique view of the 3-dimensional reconstruction of contrast-enhanced computed tomography. A MAPCA from the descending aorta was observed (**arrow**). (**C-F**) Short-axis stack of gadolinium-enhanced cardiac magnetic resonance imaging in alphabetical order from base to apex. At the base, the VSD was observed (**asterisk**), and late enhancement was prominent in the contralateral RV free wall (**arrow**). Toward the apex, late enhancement of the septum and papillary muscles on the inferior wall was prominent (**arrow**). LV, left ventricle; MAPCA, major aortopulmonary collateral artery; RV, right ventricle; VSD, ventricular septal defect.

At the age of 36 years, the patient developed frequent palpitations due to polymorphic nonsustained VTs. An implantable cardioverter defibrillator was not indicated according to the Japanese guidelines, and nonsustained VTs were not associated with syncope or sustained forms at that time.^3^ The possibility of multiple arrhythmogenic substrates led to β-blocker medication, which effectively prevented VT attacks. At the age of 42 years, she experienced her first syncopal episode after palpitations and presented urgently to our hospital. An electrocardiogram showed that the recurrent VTs were associated with syncope. Medical management with mexiletine and sotalol was attempted, but it was insufficient to suppress the VT attacks. Therefore, we evaluated the cardiac structure and possible arrhythmogenic substrates before proceeding with catheter ablation for drug-resistant VT. Echocardiography revealed preserved left ventricular (LV) function with an ejection fraction of 57%, a tricuspid regurgitant pressure gradient of 88 mm Hg without RV outflow tract flow, a large VSD, aortic override, and RV enlargement (Fig. 1A). Contrast-enhanced computed tomography showed that pulmonary perfusion was compensated by MAPCAs (Fig. 1B). Gadolinium-enhanced MRI revealed prominent late enhancement on the endocardial side throughout the RV myocardium, particularly in the free wall at the base, septum, and papillary muscles on the inferior wall (Fig. 1C-F).

## Catheter Ablation

Sedation was achieved using dexmedetomidine (0.7 μg/kg/h after a loading dose of 3 μg/kg/h) with monitoring through the Bispectral Index system (BIS; Nihon Kohden, Tokyo, Japan), regional oxygen saturation of the temporal region of the head (rSO_2_), and routine arterial blood gas examination. The hemodynamic state during intravenous anesthesia was unstable, and the baseline oxygen saturation was very low (SpO_2_, 75% and rSO_2_, 40%-50%).

Both femoral veins and the right femoral artery were used as access vessels. The ablation procedure was performed using the EnSite X EP system (Abbott, Abbott Park, IL) with the Advisor HD Grid mapping catheter (Abbott) and an open-irrigated ablation catheter (TactiFlex Contact Force Ablation Catheter, Sensor Enabled; Abbott). The procedure was conducted with a power of 40 W, a contact force of 10-20 g, a temperature limit of ≤40°C, and an irrigation rate of 13 mL/min. The activated clotting time was maintained at a minimum of 300 seconds using unfractionated heparin throughout the procedure.

**Figure 2:**
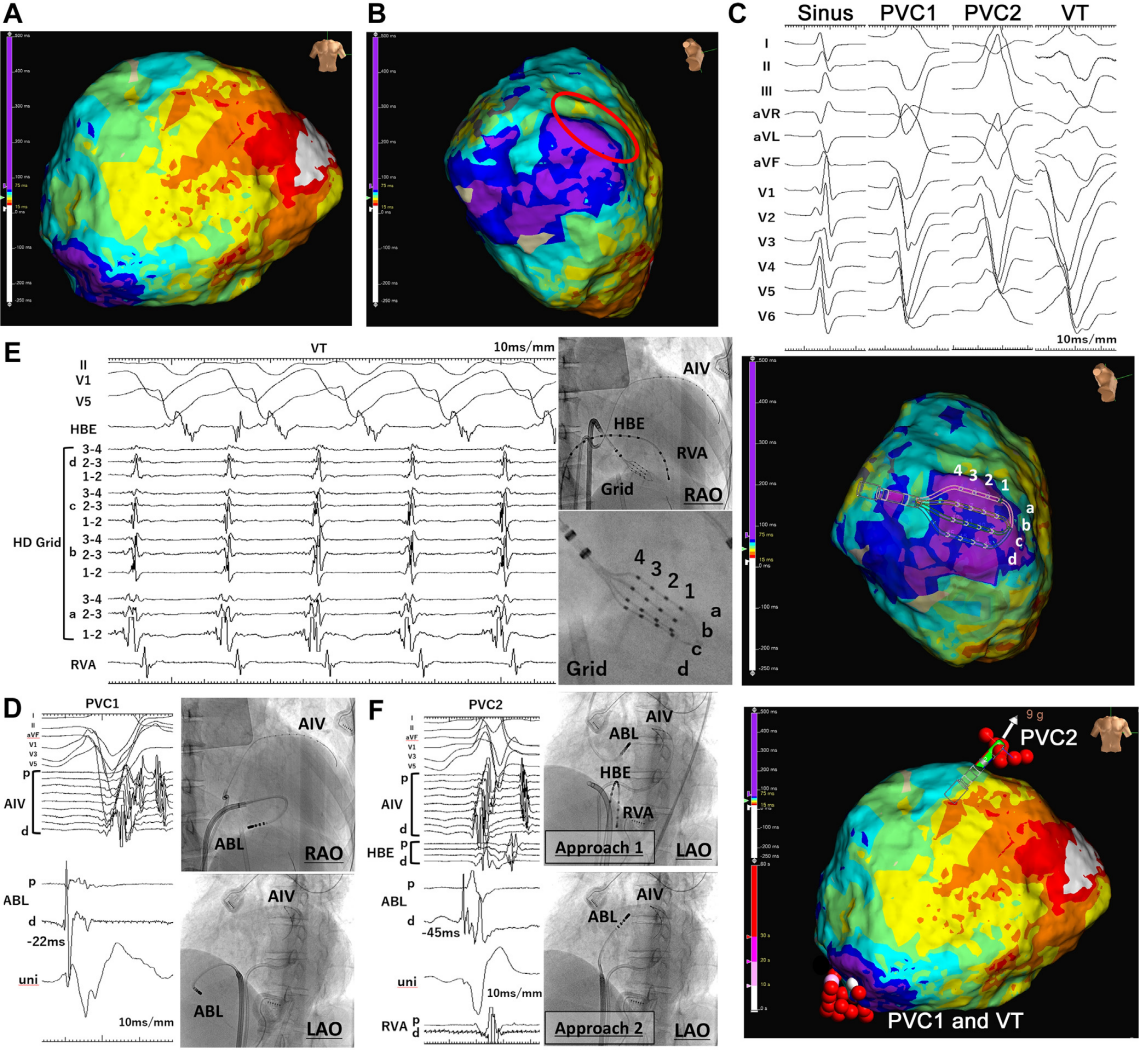
(**A**) Anteroposterior view of the RV in ILAM. (**B**) Right anterior oblique caudal view of the RV in ILAM. The isochronal crowding is circled within the red line (deceleration zone). (**C**) Twelve-lead electrocardiogram during sinus rhythm. The heart rate was 68 beats/min. The axis of PVC1 was in the superior direction, and that of PVC2 was in the inferior direction. (**D**) The local intracardiac electrocardiogram and the successful ablation site of PVC1. The red automarked points in ILAM indicate ablation sites. The local activation time preceded the QRS onset by 22 ms. (**E**) Diastolic potentials were recorded during VT using the HD grid placed in the inferolateral region of the RV. (**F**) Red automarked points in ILAM indicate ablation sites. The intracardiac electrocardiogram of the successful ablation site was 45 ms before the QRS onset of PVC2. The retrograde approach (approach 1) through the aorta was unstable because of the dilated common outflow tract and shunt flow through the VSD. The approach through the VSD (approach 2) from the RV was stable. ABL, ablation catheter potential; AIV, anterior interventricular vein potential; HBE, His bundle electrogram; ILAM, isochronal late activation mapping; LAO, left anterior oblique; PVC, premature ventricular contraction; RAO, right anterior oblique; RV, right ventricle; RVA, right ventricular apex potential; VSD, ventricular septal defect; VT, ventricular tachycardia.

First, we created a functional substrate map of the RV during sinus rhythm. Isochronal late activation mapping (ILAM) was performed using the HD Grid catheter.^4^ The mapping revealed a latest activation area adjacent to a deceleration zone, indicating abnormal conduction delay in the basal inferior free wall of the RV (Fig. 2A and B). In this case, 2 morphologies of premature ventricular contractions (PVCs) were recorded (Fig. 2C) in addition to the sustained VT. A good pace-mapping QRS morphology consistent with PVC1 was identified at a site corresponding to the delayed activation area in the ILAM. At this site, the local activation time preceded the QRS onset of PVC1 by 22 ms (Fig. 2D). Furthermore, diastolic potentials were observed during induced VT in this region, and the VT morphology resembled that of PVC1 (Fig. 2E). Entrainment pacing at this site was not feasible because of hemodynamic instability during VT. Radiofrequency application to this region successfully eliminated PVC1 and the clinical VT.

Regarding PVC2, a good pace-mapping morphology was obtained at the subvalvular position of the aortic left coronary sinus cusp (Fig. 2F), with local activation occurring 45 ms before the QRS onset of PVC2. Initially, radiofrequency energy was applied to the subvalvular region of the LV from the ablation catheter introduced through the aorta (approach 1). Although PVC2 transiently resolved, it quickly recurred because of instability of the ablation catheter caused by the dilated LV outflow tract and shunt flow through the VSD. Subsequently, a second approach was undertaken, where ablation was performed via the RV and through the VSD to reach the subvalvular region of the LV (approach 2). The catheter was advanced through a steerable sheath positioned via the VSD. The catheter’s position was adjusted by bending while maintaining coaxial alignment with the sheath, ensuring stable but not overly forceful contact with the myocardium. This VSD approach provided greater stability than the transaortic retrograde approach from above the valve. Radiofrequency applications were concluded after the clinical VT, and PVCs were successfully eliminated.

The patient underwent subcutaneous implantable cardioverter defibrillator implantation after the ablation and remained free of symptomatic VT, PVCs, and shock therapy. Notably, however, slow VT and PVC burden cannot be monitored via subcutaneous implantable cardioverter defibrillator tracings, which was a limitation in tracking the patient’s clinical course. Despite this, no ventricular arrhythmias were detected during outpatient follow-ups. More than 1 year after ablation, the patient remained free of symptomatic PVCs and VTs.

## Discussion

This case highlights 2 important clinical perspectives in the management of adult patients with unrepaired PA/VSD.

First, in patients with unrepaired PA/VSD, cumulative pressure and volume overload on the RV may contribute to the development of arrhythmogenic substrates. In this case, the critical substrate for PVC1 and VT corresponded to areas of suspected degeneration identified on late gadolinium-enhanced MRI. Diffuse fibrosis and arrhythmogenic substrates are known to be associated with RV remodeling in patients with repaired tetralogy of Fallot.^5^ Similarly, local myocardial degeneration in the RV free wall due to shunt flow through the VSD may contribute to diffuse fibrosis and the formation of arrhythmogenic substrates.^6^ Preoperative gadolinium-enhanced MRI proved valuable in identifying potential sites of degeneration likely linked to arrhythmogenic substrates.

Second, when performing VT ablation in patients with CHD who have respiratory and hemodynamic instability, a patient-specific strategy is essential. Functional substrate mapping, such as ILAM, enables the stable and safe assessment of arrhythmic substrates during sinus rhythm, even in high-risk cases. In this patient, retrograde catheter insertion through the aorta was unstable because of the dilated LV outflow tract and shunt flow through the VSD. Paradoxically, however, stable catheter manipulation was achieved using a subaortic valvular approach from the RV through the VSD. Focal ventricular arrhythmias reportedly account for 14.3% of all ventricular arrhythmias in patients with CHD, with significant variability in their origins.^7^ Therefore, safe and flexible approaches tailored to address multisource PVCs should be considered.

## Conclusion

We successfully managed a case of drug-resistant VT in an adult patient with unrepaired PA/VSD. Preoperative assessment using cardiac MRI and a patient-specific ablation strategy enabled effective clinical management, even in the context of unrepaired complex CHD.

**Novel Teaching Points**

• The substrate of ventricular tachycardia in unrepaired pulmonary atresia/ventricular septal defect (VSD) was located in areas of myocardial degeneration, likely associated with the shunt flow through the unrepaired VSD.
• An approach through the unrepaired VSD proved effective in eliminating premature ventricular contractions originating from the dilated left ventricular outflow tract. An individualized strategy tailored to the patient’s specific anatomic features is essential for determining the optimal approach route.
